# Performance Evaluation of MEMS-Based Automotive LiDAR Sensor and Its Simulation Model as per ASTM E3125-17 Standard

**DOI:** 10.3390/s23063113

**Published:** 2023-03-14

**Authors:** Arsalan Haider, Yongjae Cho, Marcell Pigniczki, Michael H. Köhler, Lukas Haas, Ludwig Kastner, Maximilian Fink, Michael Schardt, Yannik Cichy, Shotaro Koyama, Thomas Zeh, Tim Poguntke, Hideo Inoue, Martin Jakobi, Alexander W. Koch

**Affiliations:** 1 IFM—Institute for Advanced Driver Assistance Systems and Connected Mobility, Kempten University of Applied Sciences, Junkersstrasse 1A, 87734 Benningen, Germany; 2 Institute for Measurement Systems and Sensor Technology, Technical University of Munich, Theresienstr. 90, 80333 Munich, Germany; 3 Blickfeld GmbH, Barthstr. 12, 80339 Munich, Germany; 4 IPG Automotive GmbH, Bannwaldallee 60, 76185 Karlsruhe, Germany; 5 Department of Vehicle System Engineering, Kanagawa Institute of Technology, Shimoogino 1030, Atsugi 243-0292, Kanagawa, Japan

**Keywords:** micro-electro-mechanical systems, automotive LiDAR sensor, ASTM E3125-17 standard, advanced driver-assistance system, open simulation interface, functional mock-up interface, functional mock-up unit, point-to-point distance tests, user-selected tests, proving ground, PointPillars

## Abstract

Measurement performance evaluation of real and virtual automotive light detection and ranging (LiDAR) sensors is an active area of research. However, no commonly accepted automotive standards, metrics, or criteria exist to evaluate their measurement performance. ASTM International released the ASTM E3125-17 standard for the operational performance evaluation of 3D imaging systems commonly referred to as terrestrial laser scanners (TLS). This standard defines the specifications and static test procedures to evaluate the 3D imaging and point-to-point distance measurement performance of TLS. In this work, we have assessed the 3D imaging and point-to-point distance estimation performance of a commercial micro-electro-mechanical system (MEMS)-based automotive LiDAR sensor and its simulation model according to the test procedures defined in this standard. The static tests were performed in a laboratory environment. In addition, a subset of static tests was also performed at the proving ground in natural environmental conditions to determine the 3D imaging and point-to-point distance measurement performance of the real LiDAR sensor. In addition, real scenarios and environmental conditions were replicated in the virtual environment of a commercial software to verify the LiDAR model’s working performance. The evaluation results show that the LiDAR sensor and its simulation model under analysis pass all the tests specified in the ASTM E3125-17 standard. This standard helps to understand whether sensor measurement errors are due to internal or external influences. We have also shown that the 3D imaging and point-to-point distance estimation performance of LiDAR sensors significantly impacts the working performance of the object recognition algorithm. That is why this standard can be beneficial in validating automotive real and virtual LiDAR sensors, at least in the early stage of development. Furthermore, the simulation and real measurements show good agreement on the point cloud and object recognition levels.

## 1. Introduction

**Figure 1:**
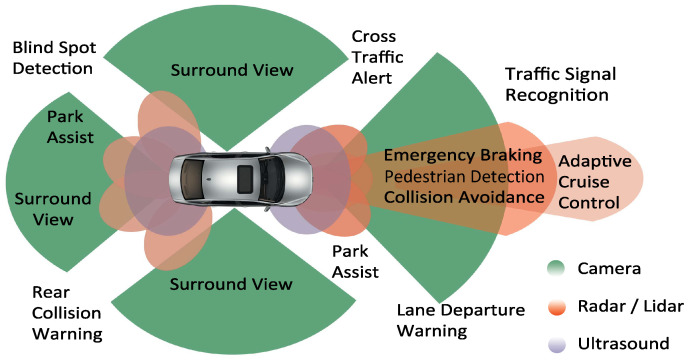
ADAS used in modern vehicles, source: adapted with permission from [5].

The advanced driving-assistance system (ADAS) increases vehicle and road safety. Car manufacturers are installing ADAS in modern cars to enhance driver safety and comfort, as shown in Figure 1. ADAS acquires the vehicle’s surrounding information from the environmental perception sensors [1]. Light detection and ranging (LiDAR), radio detection and ranging (RADAR), cameras, and ultrasonics are the current sensing technologies used for ADAS [2]. As these sensors support human drivers, their measurement performance must be verified before they are installed in the vehicles. However, the complexity of ADAS is also increasing over time. The validation of such a complex system in the real world is not feasible due to the cost and timing constraints [3].

Therefore, the automotive industry has started considering type approval based on virtual tests, requiring virtual ADAS and environmental perception sensors [4]. Consequently, the virtual environmental perception sensor’s operational performance also needs to be verified before using them for the virtual validation of ADAS.

LiDAR sensors have gained significant attention for ADAS over the last few years due to their wider field of view (FoV) and higher ranging accuracy compared to RADAR [6]. Therefore, car manufacturers install them for autonomous vehicle prototyping [7,8,9]. As a result, LiDAR sensors have become a crucial sensor technology for the ADAS perception system. Hence, their performance evaluation is necessary before using them for ADAS.

Several methods are described in the literature to characterize the performance of LiDAR sensors [10,11,12,13,14,15,16,17,18,19]. However, no established uniform test standard is currently available for the operational performance assessment of the real and virtual automotive LiDAR sensors. Although, this year, two projects [20,21] have been initiated to develop specifications and testing frameworks for the performance evaluation of automotive LiDAR sensors. However, these standards still need to be released. ASTM International released a detailed standardized documentary test procedure E3125-17 to evaluate medium-range laser scanners’ point-to-point distance measurement performance. A medium-range system can measure the distance within the range of 2 m to 150 m. According to this standard, the LiDAR manufacturer should specify the size, material, and optics characteristics of sphere and plate targets that can yield a minimum of 300 and 100 LiDAR points so that the dimensions or size of the targets can be easily estimated from the obtained LiDAR points. Furthermore, the LiDAR sensor-derived point-to-point distance measurement error $d_{error}$ should not exceed the maximum permissible error (MPE) specified by the manufacturer, which is 20 mm in this case. A derived point is obtained by processing a set of points from the target surface, representing the target’s center. This standard also helps to understand whether sensor measurement errors are due to internal or external influences. This test standard is recommended for spherical coordinate 3D imaging systems but can also be applied to non-spherical coordinate systems [22].

Therefore, in this paper, we have evaluated the 3D imaging and point-to-point distance measurement performance of the Blickfeld micro-electro-mechanical systems (MEMS)-based automotive LiDAR sensor and its simulation model (LiDAR FMU) according to the ASTM E3125-17 tests method. It should be noted that same authors have developed the LiDAR FMU model in their previous work [23] for the simulation-based testing of ADAS, and the presented paper is the continuity of it. The authors want to bring the scientific community’s attention, focusing on developing and validating automotive real and virtual LiDAR sensors to the ASTM E3125-17 standard. The test cases defined in this standard are easy to implement and can help to evaluate the measurement performance of automotive real and virtual LiDAR sensors, at least in the early stage of development. The first realization of this standard for the performance evaluation of terrestrial laser scanners (TLS) was reported in [24]. To the author’s knowledge, this is the first research paper evaluating real and virtual MEMS-based automotive LiDAR sensor operational performance according to ASTM E3125-17 standard test procedures.

The LiDAR sensor model is developed as a functional mock-up unit (FMU) by using the standardized open simulation interface (OSI) and functional mock-up interface (FMI) [23]. The model is packaged as an OSI sensor model packaging (OSMP) FMU [25]. Therefore, it was integrated successfully into the co-simulation environment of CarMaker from IPG Automotive. The virtual LiDAR sensor considers the accurate modeling of scan pattern and complete signal processing toolchain of the Cube 1 LiDAR sensor, as described in [23].

This paper is structured as follows: Section 2 describes the LiDAR sensor background. The specifications of the devices under test are given in Section 3. The ASTM E3125-17 standard test methods overview is discussed in Section 4, and the data analysis methodologies of the tests are described in Section 5. In addition, Section 6 provides a detailed description of the test setup and results. Finally, Section 7 and Section 8 provide the conclusion and outlook, respectively.

## 2. Background

LiDAR sensors can be classified as scanning and non-scanning LiDARs [26]. Flash LiDAR sensors are the non-scanning type of LiDAR. They can measure distances up to 50 m and are suitable for ADAS safety applications, including blind-spot detection (BSD) and forward collision warning (FCW) [27]. On the other hand, scanning LiDAR sensors consist of optical phased array (OPA) scanners, mechanical rotating scanners, or MEMS scanners [26]. These sensors can detect objects up to 250 m and can be used for the lane departure warning system (LDWS), adaptive cruise control (ACC), BSD, and FCW [27,28]. Specifically, MEMS LiDAR sensors are getting more attention for automotive applications because they are small, lightweight, and power efficient [29]. Furthermore, MEMS-based LiDAR sensors are also used for agricultural, archaeological surveys, and crowd analytics [26,30]. The following section will discuss the MEMS-based LiDAR sensors working principle in detail.

### Working Principle of MEMS LiDAR Sensor

**Figure 2:**
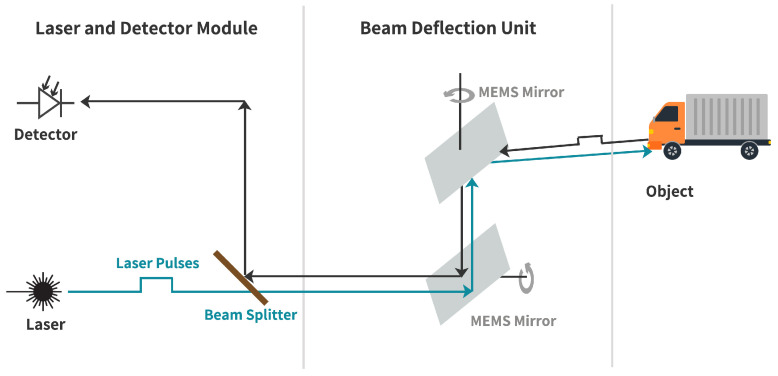
Block diagram of MEMS LiDAR sensor, source: adapted with permission from [31].

MEMS-based LiDAR consists of a laser and detector module (LDM) and beam deflection unit (MEMS mirrors), as shown in Figure 2. The laser source emits laser pulses, and the beam deflection unit deflects the beam in different directions to obtain holistic imaging of the environment. The photodetector receives the laser light partly reflected from the target’s surface. It measures the round trip delay time (RTDT) τ that laser light takes to hit an object and return to the detector. With the RTDT τ, the range *R* can be calculated as [23]:

(1) $$R=\frac{c\;\cdot \;\tau }{2},$$

where the range is denoted by *R*, *c* is the speed of light, and τ is the RTDT, also known as the time of flight (ToF).

## 3. Devices Under Test

**Figure 3:**
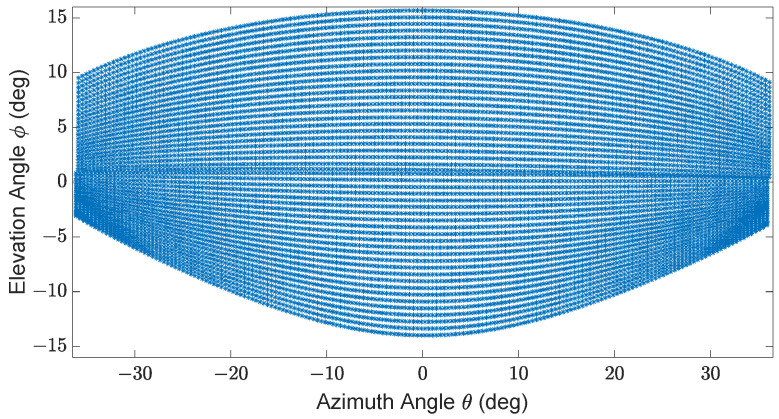
Exemplary elliptical shape scan pattern of Cube 1. Specifications: $\pm 36^{\circ }$ horizontal and $\pm 15^{\circ }$ vertical FoV, 50 scan lines, $0.4^{\circ }$ horizontal angle spacing, frame rate 5.4 Hz, the maximum detection range is 250 m, and the minimum detection range is 1.5 m.

In the context of this work, we have evaluated the 3D imaging and point-to-point distance estimation performance of Blickfeld Cube 1 and its simulation model LiDAR FMU. Cube 1 was designed for industrial applications but is also used for automotive applications. Cube 1 comprises a single laser source and a beam deflection unit, so-called mirrors. The mirrors deflect the laser beam using two 1D MEMS mirrors oriented horizontally and vertically, having a phase difference of $45^{\circ }$, and it generates the elliptical shape scan pattern illustrated in Figure 3 [32,33]. In addition, the specifications of the LiDAR sensor are listed in Table 1.

### LiDAR FMU Model

**Figure 4:**
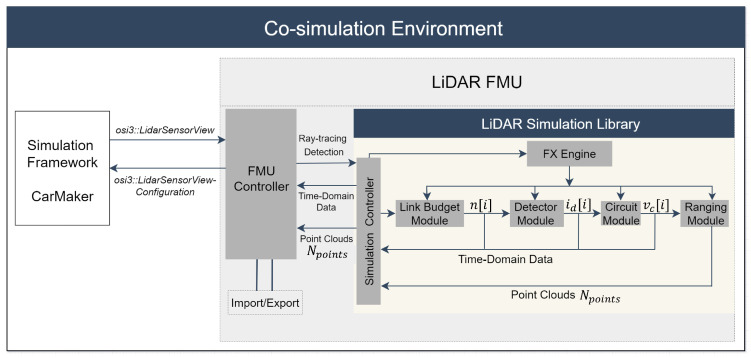
Co-simulation framework of the LiDAR FMU model [23].

This section will provide an overview of the toolchain and signal processing steps of the LiDAR FMU model. A detailed description of the LiDAR FMU modeling methodology can be found in [23]. The toolchain and signal processing steps of the LiDAR FMU model are shown in Figure 4. As mentioned in Section 1, the model is built as an OSMP FMU. It uses the virtual environment and ray tracing module of CarMaker.

The material properties of the simulated objects are specified in the material library of CarMaker. The test scenarios are generated in the virtual environment of CarMaker. After that, the FMU controller calls the LiDAR Simulation Library and passes the required input configuration via *osi3::LidarSensorViewConfiguration* to CarMaker. CarMaker verifies the input configuration and passes the ray tracing data via *osi3::LidarSensorView::reflection* to LiDAR FMU for further processing. The simulation controller is the main component of the LiDAR Simulation Library. It provides the interaction with the library, for instance, configuring the simulation pipeline, inserting ray tracing data, executing the simulation’s steps, and retrieving the results. The Link Budget Module calculates the photons over time n[i]. The Detector Module captures these photons’ arrivals and converts them into a photocurrent signal $i_{d}\left[i\right]$. In the next step, the Circuit Module amplifies the photocurrent signal $i_{d}\left[t\right]$ of the detector to a voltage signal $v_{c}\left[i\right]$, which is processed by the Ranging Module. The Ranging Module determines the range and intensity of the target based on the $v_{c}\left[i\right]$ received from the analog circuit for every reflected scan point. The Effect Engine (FX engine) includes a series of interfaces that interact with environmental or sensor-specific imperfections and simulation blocks [23]. The sensor model specifications are the same as Cube 1 given in Table 1, and it uses the same scan pattern as the real sensor shown in Figure 3.

## 4. ASTM E3125-17

This standard defines the specifications and test procedures to evaluate the measurement performance of medium-range 3D imaging systems that produce point clouds of an object of interest. Two types of tests are defined in this standard: two-face tests and point-to-point distance tests to evaluate the measurement performance of the device under test (DUT) [22]. Two-face tests are recommended for 3D-imaging systems that simultaneously measure the target in front and back modes. However, these tests are not applicable in this use case because Cube 1 can only measure in front mode. The point-to-point distance tests involve the measurements of target distances using the DUT and reference instrument (RI) in various orientations and positions within the DUT measurement capability. These tests also provide the impact of mechanical and optical misalignment within the DUT on the measured distance to the target [22].

### 4.1. Specification of Targets

**Figure 5:**
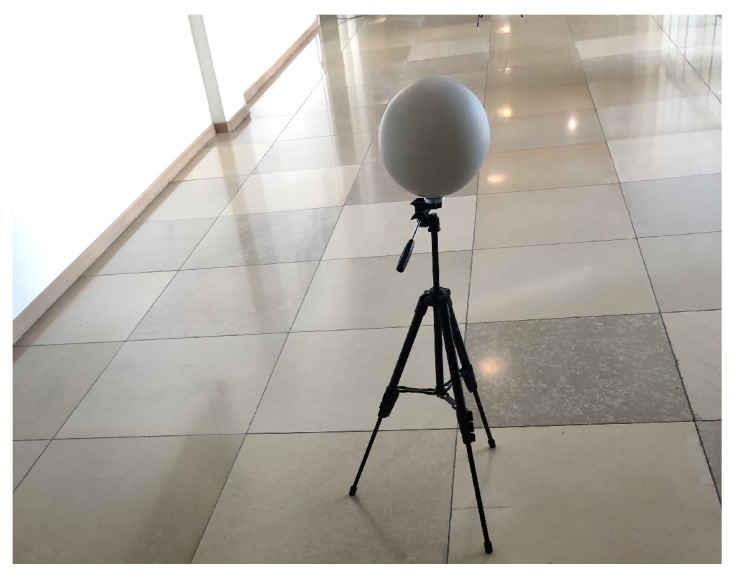
The sphere target is made of plastic with a special matt-textured varnish. It also has a removable magnetic base (M8 thread).

**Figure 6:**
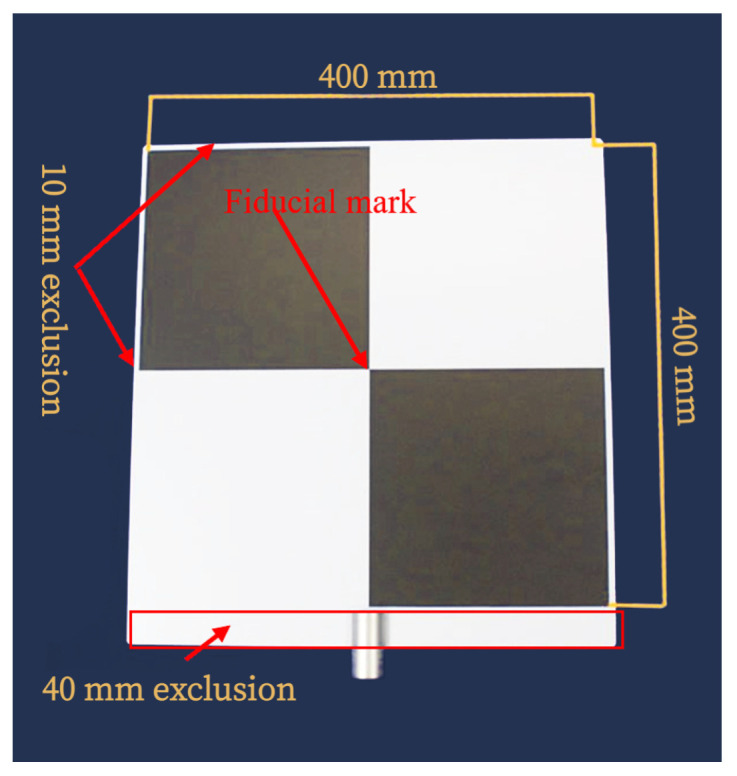
The rectangular laser scanner checkerboard has an area of 450 mm × 420 mm with a 1/4 inch adapter. As required in the standard, the LiDAR points from the edges of the plate target should not be considered for the point-to-point distance measurement. That is why the exclusion region is defined for the plate target. As a result, the active area of the plate target becomes 400 mm × 400 mm. In addition, the fiducial mark is defined at the center of the plate target so the RI can aim directly at it for the reference distance measurement.

This standard specifies the sphere and plate as targets for point-to-point distance tests. The sphere target is used for all point-to-point distance tests except relative range tests that use the plate target. According to the ASTM standard, the type of target materials, including their optical characteristics, should be specified by the DUT manufacturer because the material and optical properties influence the measurement. Users can choose any material’s targets if the DUT manufacturer does not specify them. Moreover, the sphere and plate target should be big enough to yield a minimum of 300 and 100 measured points after point selections so that the size or the dimensions of the targets can be estimated from the LiDAR point clouds. In this work, we use a diffuse reflective sphere target made up of plastic, as shown in Figure 5. The manufacturer has specified a 200.0 mm diameter of the sphere target with an uncertainty of ± 1 mm. However, we have conducted several measurements in the lab and measured a 200.9 mm diameter of the sphere target in the lab with a Hexagon Romer Absolute Arm 7525 SI [35] with a confidence score of 95%. In addition, we use the rectangular laser scanner checkerboard, as shown in Figure 6, for the relative range tests.

### 4.2. Inside Test

**Figure 7:**
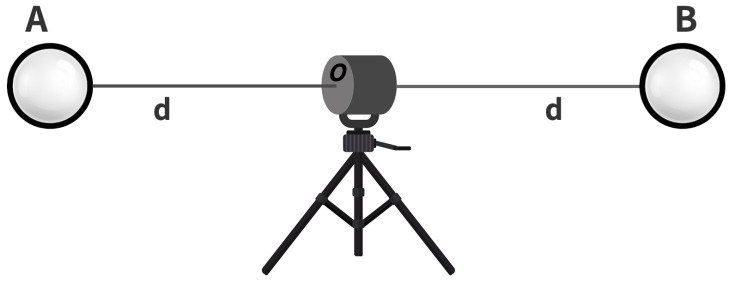
Inside Test layout. The distance *d* of both spheres A and B from the DUT shall be equal. The manufacturer should specify the distance *d*; if in case they do not specify it, the user can choose any value of distance.

This test involves the distance measurement between two sphere targets placed equidistant (d) to the DUT and whose centers are nominally collinear with the DUT origin, as shown in Figure 7. The center of the spheres should be aligned so that the azimuth angle of sphere A is $\theta_{a}$ from the DUT, then sphere B’s azimuth angle should be within $\theta_{a}+180^{\circ }\pm 10^{\circ }$. The sphere targets and sensor height shall be the same, so the elevation angle of spheres A and B should be within $0^{\circ }\pm 10^{\circ }$. The DUT should scan objects in both front-sight and back-sight modes. If the DUT can measure only in front-sight mode, the user can measure both targets in front-sight mode, but the sensor needs to be rotated to measure the back sphere [22].

### 4.3. Symmetric Test

**Figure 8:**
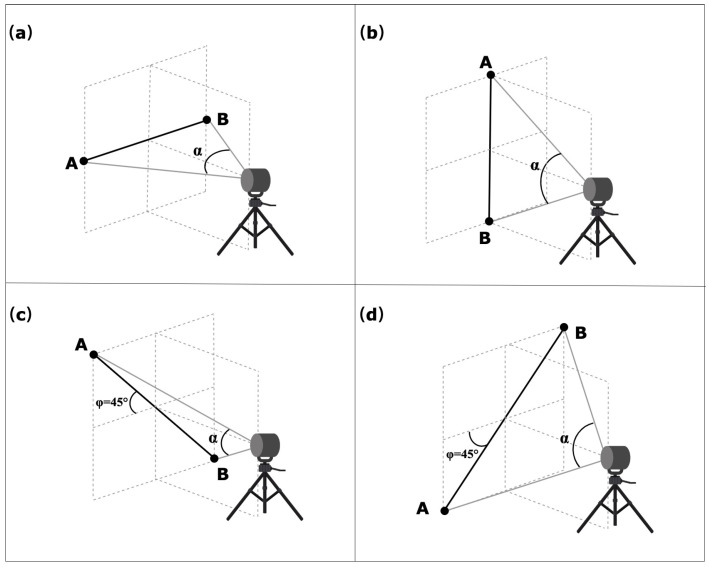
Measurement method of the symmetric tests for the sphere targets A and B placed in orientations (**a**–**d**). α is an angular sweep between two targets, and φ is the angle between the bar and plane, source: adapted with permission from [22].

This test involves the point-to-point distance measurement of sphere targets placed symmetrically to the DUT in different orientations and positions, as shown in Figure 8. These tests can be realized with the two azimuth angles ($\theta =0^{\circ }$ and $\theta +90^{\circ }\pm 10^{\circ }$) between the DUT and the center of line AB. This test involves eight measurements either in front-sight mode or in back-sight mode. The user can select any distances between the pairs of spheres and distances from the DUT that meets the angular sweep α requirements mentioned in the standard, and those values should be within the work volume of the DUT. The angular sweep between the sphere targets shall be at least $80^{\circ }$ in the plane containing the target’s center and DUT origin [22].

### 4.4. Asymmetric Test

**Figure 9:**
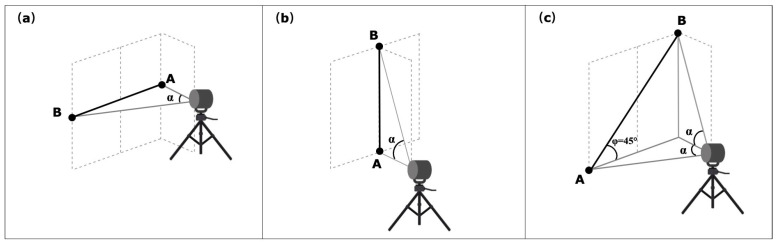
The layout of asymmetric tests for the sphere targets A and B placed in orientations (**a**–**c**). α is an angular sweep between two targets, and φ is the angle between the bar and plane, source: adapted with permission from [22].

This test involves the point-to-point distance measurement of sphere targets placed asymmetrically concerning the DUT in different orientations and positions, as shown in Figure 9. This test is similar to the symmetric test, but the placement of the sphere targets is different. This test involves six measurements with the two orientations of DUT at azimuth angle $\theta =0^{\circ }$ and $\theta +90^{\circ }\pm 10^{\circ }$. The user can select any distances between the pairs of spheres and distances from the DUT that meet the angular sweep α requirements mentioned in the standard, and those values should be within the work volume of the DUT. However, the angular sweep α between the pairs of spheres target shall be at least $40^{\circ }$.

### 4.5. Relative Range Test

**Figure 10:**
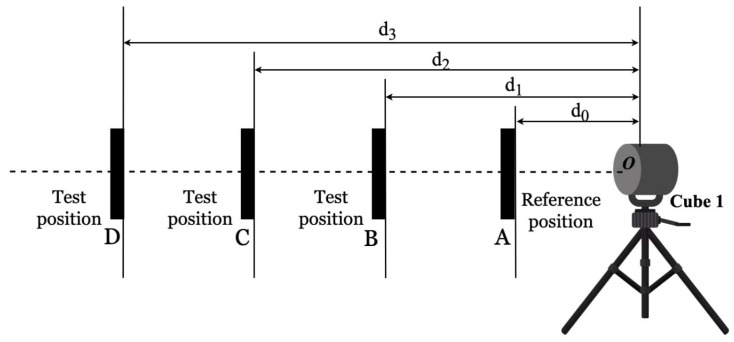
Layout of relative range test, source: adapted from [24].

The relative-range test involves the distance measurement of a plate target at a reference position and three different test positions (AB, AC, AD) in a front-sight mode, as shown in Figure 10. The center of the planar target and the origin of the DUT should be collinear. The reference distance of the DUT should be specified by the manufacturer. If this is not the case, then the user is allowed to choose any distance within the work volume of the DUT [22].

### 4.6. User-Selected Tests

**Figure 11:**
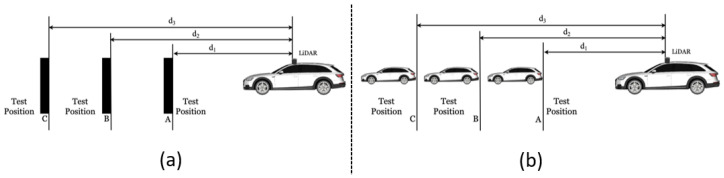
(**a**) Layout of user-selected tests for 10% reflective planar Lambertian target. (**b**) Layout of user-selected tests for the vehicle target.

The standard requires two additional user-selected tests to complete the point-to-point distance measurement tests. Again, the sphere or the planar object can be used as the target. We are using a DUT for automotive applications, which is why we use a vehicle and a 10% reflective Lambertian plate as targets for these tests. It should be noted that we measured the reflectivity of the real vehicle before using it for these tests. Furthermore, the measurements are performed at the Jtown proving ground [36] because we want to validate the measurement performance of the DUT in sunlight. The DUT was mounted on the test vehicle’s roof, as shown in Figure 11.

It should be noted that inside, symmetric, asymmetric, and relative range tests are performed in the lab to evaluate the 3D imaging and distance measurement performance of Cube 1 in the lab. The sensor origin and target center are collinear for these tests. But, the DUT was placed on the vehicle roof for user-selected tests, and the sensor origin is not collinear with the target center.

## 5. Data Analysis

The ASTM standard introduces a calculation method to find the coordinates of the derived points for sphere and plate targets. This process is repetitive; thus, Python and MATLAB-based programs for data analysis, filtration, and least square fit are used.

### 5.1. Calculation of Sphere Target Derived Points Coordinates

**Figure 12:**
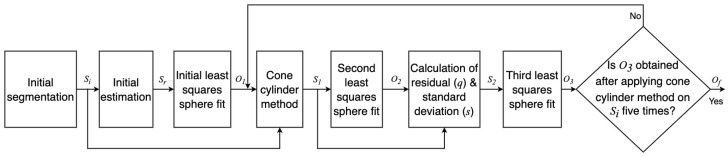
Procedure to calculate sphere-derived point coordinates.

The ASTM E3125-17 standard has introduced a procedure to calculate the derived point for a sphere target, as shown in Figure 12.

**Figure 13:**
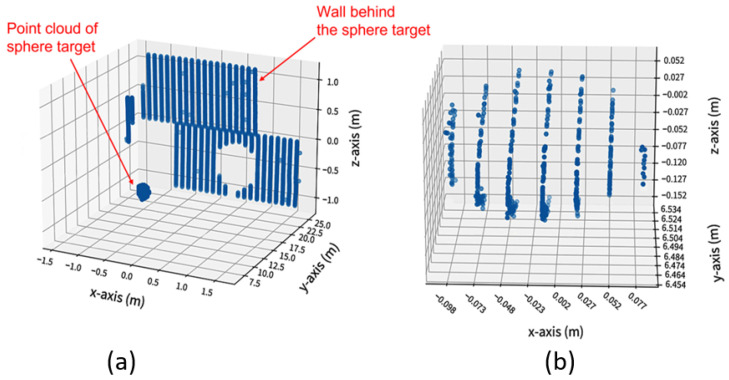
(**a**) Exemplary raw point cloud data from every object in the FoV of DUT. (**b**) Segmented data representing point cloud $S_{i}$ of sphere target.

**Figure 14:**
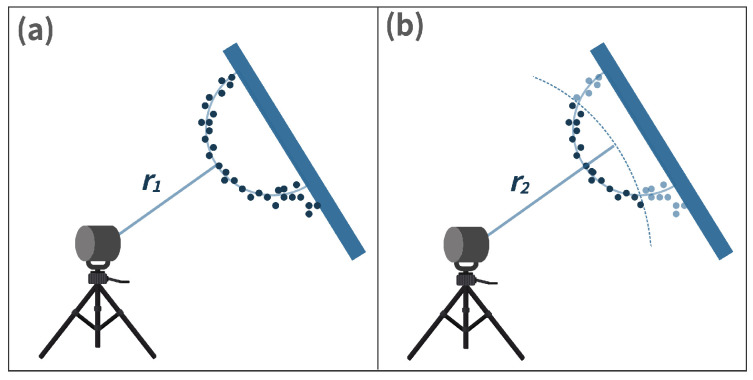
Closest point method. (**a**) $r_{1}$ is the median of the M smallest distances of points from the DUT origin. (**b**) $r_{2}=r_{1}+\frac{R}{2}$, where R denotes the radius of the sphere target, source: reproduced with permission from [22].

**Figure 15:**
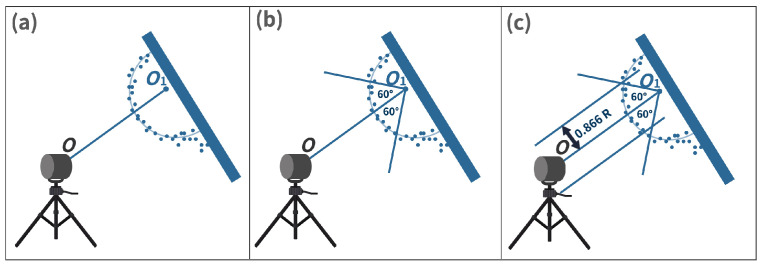
Cone cylinder method. (**a**) A straight line $O_{1}O$ is drawn between the origin of the DUT and the initial derived point. (**b**) A cone with an apex located at $O_{1}$ with an opening angle of $120{\;}^{\circ }$ is constructed. (**c**) A cylinder collinear to $O_{1}O$ with 0.866R is drawn, source: reproduced with permission from [22].

**Figure 16:**
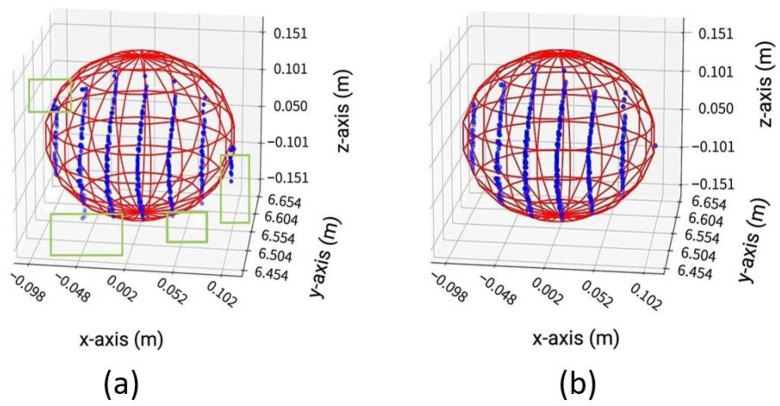
Comparison between sphere’s point clouds after initial $S_{i}$ and final $S_{f}$ LSSF. (**a**) Sphere point cloud after initial LSSF $S_{i}$. (**b**) Sphere point cloud after final LSSF $S_{f}$. The initial LSSF $S_{i}$ contains 381 points, and a 201.4 mm sphere diameter ∅ is estimated from it. The final LSSF $S_{f}$ contains 306 points, and a 201.2mm sphere diameter ∅ is estimated from it.

1. Initial segmentation: The measured data corresponding to sphere targets shall be segmented from the surroundings since the DUT measures every object in its work volume [22]. The exemplary point clouds before and after the initial segmentation are shown in Figure 13. The points obtained after the initial segmentation are regarded as $S_{i}$.
2. Initial estimation: The initial estimation is used to find the coordinate of the derived point, which is the center of the point set $S_{i}$ received from the surface of the sphere target [22]. Several methods are introduced in the standard for the initial estimation, including manual estimation, the software provided by the DUT manufacturer, and the closest point method [22]. In this work, we have used the closest point method to estimate the derived point, as shown in Figure 14. First, the Euclidean distances of all the LiDAR points in data set $S_{i}$ to the origin of DUT are calculated. $r_{1}$ is determined as the median of the M closest distances of points from the DUT origin, as shown in Figure 14a). Afterward, the $r_{2}$ distance is calculated by adding the half radius R/2 of the sphere target to $r_{1}$, as illustrated in Figure 14b). The points within the radius $r_{2}$ are represented by $S_{r}$ [22].
3. Initial least squares sphere fit: A non-linear, orthogonal, least squares sphere fit (LSSF) is used on the $S_{r}$ points to determine the initial derived point $O_{1}$. The general equation of the sphere can be expressed as follows [37]:
  (2) $${\left(x-x_{c}\right)}^{2}+{\left(y-y_{c}\right)}^{2}+{\left(z-z_{c}\right)}^{2}=R^{2},$$
  where *x*, *y*, and *z* are the cartesian coordinates of the points on the sphere’s surface, $\left(x_{c},y_{c},z_{c}\right)$ and *R* represent the center and radius of the sphere, respectively. Equation (2) is expanded and rearranged as [37]
  (3) $$x^{2}+y^{2}+z^{2}=2xx_{c}+2yy_{c}+2zz_{c}+R^{2}-x_{c}^{2}-y_{c}^{2}-z_{c}^{2}.$$
  To apply the least squares fit on all points obtained from the sphere surface, Equation (3) can be expressed in vector/matrix notation for all points in the data set as given in [37]
  (4) $$\vec{f}=\left[\begin{array}{c} x_{i}^{2}+y_{i}^{2}+z_{i}^{2}\\ x_{i+1}^{2}+y_{i+1}^{2}+z_{i+1}^{2}\\ \vdots \\ x_{n}^{2}+y_{n}^{2}+z_{n}^{2} \end{array}\right],$$
  (5) $$A=\left[\begin{array}{cccc} 2x_{i} & 2y_{i} & 2z_{i} & 1\\ 2x_{i+1} & 2y_{i+1} & 2z_{i+1} & 1\\ \vdots & \vdots & \vdots & \vdots \\ 2x_{n} & 2y_{n} & 2z_{n} & 1 \end{array}\right],$$
  (6) $$\vec{c}=\left[\begin{array}{c} x_{c}\\ y_{c}\\ z_{c}\\ R^{2}-x_{c}^{2}-y_{c}^{2}-z_{c}^{2} \end{array}\right],$$
  (7) $$\vec{f}=A\vec{c}.$$
  Here, the terms $x_{i}$, $y_{i}$, and $z_{i}$ represent the initial points of the data set, and $x_{n}$, $y_{n}$, and $z_{n}$ show the last points of the data set. Vector $\vec{f}$, matrix *A*, and vector $\vec{c}$ contain the expanded terms of the sphere, Equation (3). The vector $\vec{f}$ is the least squares fit method used to calculate the vector $\vec{c}$ that contains the sphere’s center coordinates and radius *R*. We used the Python Numpy library [38] least squares function to calculate the vector $\vec{c}$ that returns the sphere’s center $O_{1}$ coordinates and radius *R*. We can fit a sphere to our original data set by using the output of vector $\vec{c}$.
4. Cone cylinder method: As recommended in the standard, in the next step, we refine the derived point $O_{1}$ coordinates through the cone cylinder method for the sphere target, as shown in Figure 15. A straight line $O_{1}O$ is drawn between the origin of DUT *O* and the initial derived point $O_{1}$ given in Figure 15a. A new point data set $S_{1}$ is generated from the initial segmented points $S_{i}$, which lie within both cones shown in Figure 15b,c [22].
5. Second least squares sphere fit: Furthermore, an orthogonal non-linear LSSF is applied to the $S_{1}$ data set to find the updated derived point $O_{2}$ of the sphere target [22].
6. Calculation of residuals and standard deviation: Afterward, the residual and standard deviation of every point within $S_{1}$ is calculated. The residual is the difference between the sphere-updated derived point $O_{2}$ and the points in the set $S_{1}$. In the next step, a new point set $S_{2}$ is defined, including the points whose absolute residual value is less than three times the standard deviation [22].
7. Third least squares sphere fit: On the new set $S_{2}$, another LSSF is performed to find the updated derived point $O_{3}$ [22].
8. Calculation of final derived point coordinates: The final derived point $O_{f}$ is determined after at least four more times repeating the previous procedures on $S_{i}$ as recommended in the standard. The newly derived point $O_{3}$ of the prior task is regarded as $O_{1}$ in the subsequent iteration tasks [22]. The comparison between the sphere’s point cloud after initial $S_{i}$ and final $S_{f}$ LSSF is given in Figure 16.

#### Test Acceptance Criteria

The distance between the initial estimation $O_{1}$ and the final derived point $O_{f}$ shall be less than 20% of the nominal radius of the sphere target, that is, 100.45 mm × 0.2 = 20.09 mm; otherwise, another initial estimation should be conducted [22].

1. According to the specifications of the DUT, the value of the distance MPE is equal to 20 mm. The distance error $d_{error}$ shall be less than 20 mm [22]. The distance error $d_{error}$ between the two derived points can be written as:
  (8) $$d_{error}=d_{meas}-d_{ref}.$$
  (9) $$d_{meas}=\sqrt{{\left(x_{t}-x_{s}\right)}^{2}+{\left(y_{t}-y_{s}\right)}^{2}+{\left(z_{t}-z_{s}\right)}^{2}},$$
  where the targets’ *x*, *y*, and *z* coordinates are denoted by the subscript *t* and the sensors’ by *s* [39].
  (10) $$d_{ref}=\sqrt{{\left(x_{t}-x_{RI}\right)}^{2}+{\left(y_{t}-y_{RI}\right)}^{2}+{\left(z_{t}-z_{RI}\right)}^{2}},$$
  where the targets’ *x*, *y*, and *z* coordinates are denoted by the subscript *t* and the reference instrument by RI [39]. We have used Leica DISTO S910 as the RI for the real measurements, and OSI ground truth interface *osi3::GroundTruth* is used to retrieve the sensor’s origin and target center position in 3D coordinates in the virtual environment. [40,41].
2. In the case of the sphere target, the number of points in the $S_{2}$ data set shall be greater than 300 [22].

### 5.2. Calculating Coordinates of Derived Point for the Plate Target

**Figure 17:**
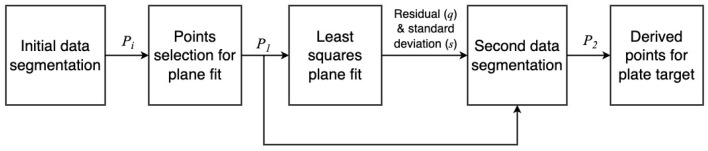
Procedure to calculate plate-derived point coordinates.

The standard has introduced a procedure to calculate the derived point for a plate target. Figure 17 shows the steps to calculate the derived point for the plate target.

**Figure 18:**
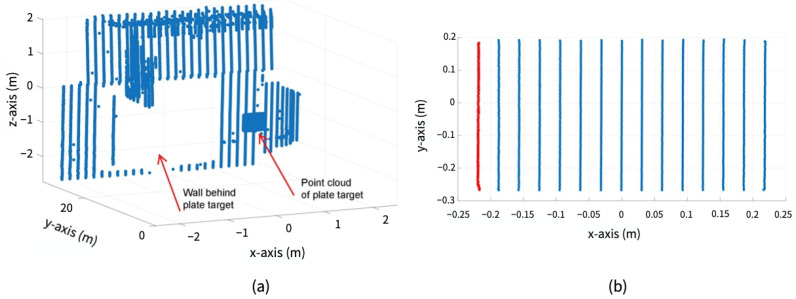
Initial data segmentation. (**a**) Raw point cloud data from every object within the FoV of DUT. (**b**) Refined data $P_{i}$ representing point cloud of plate target. The red dotted points are removed from the edges of the rectangular plate as the standard recommends. The effective width *W* and length *L* become 400 mm × 400 mm.

1. Initial data segmentation: The DUT provides point clouds from all the objects within its work volume. Therefore, all points received from the objects of no interest need to be filtered, as shown in Figure 18. After initial segmentation, the rest of the points are regarded as the points set $P_{i}$ [22].
2. Point selection for plane fit: Afterward, as required in the standard, the measured points from the edges of a rectangular plate are removed to fit a plane. This new point set is designated as $P_{1}$ [22].
3. Least squares plane fit: The least squares plane fit (LSPF) method is applied on the point set $P_{1}$ defined in [42] to determine the location and orientation of the plate target. In addition, the standard deviation *s* of residual *q* of the plane fit is measured at each position of the plate target, as required in the standard. The plane fit residuals *q* are the orthogonal distances of every measured point of the plate target to its respective plane [22].
4. Second data segmentation: The points whose residuals *q* are greater than double the corresponding standard deviation *s* were eliminated to visualize the best plane fit, as suggested in the standard. The updated point set is regarded as $P_{2}$. The number of points in $P_{2}$ should be more than 95% of all measured points from the plate target. The distance error $d_{error}$ and the root mean square (RMS) dispersion of the residuals *q* in $P_{2}$ are calculated using Equation (11) at the reference and each test position.
  (11) $$q_{rms}=\sqrt{\frac{\sum_{j=1}^{n}q_{j}^{2}}{N}},$$
  where *q* is the residual, and *N* denotes the number of points in the subset $P_{2}$ [22].
5. Derived point for plate target: Although the plate target has a fiducial mark, it was still challenging to determine a derived point precisely at the center of the plate target. Because of that, we use the 3D geometric center method on the point set $P_{2}$ to determine the derived point of the plate target, as recommended in the standard [22].

#### Test Acceptance Criteria

1. The distance error $d_{error}$ shall be less than 20 mm, and it can be calculated with Equation (8) [22].
2. The plate target should yield a minimum of 100 points in the point cloud [22].

## 6. Tests Setup and Results

### 6.1. Inside Test

**Figure 19:**
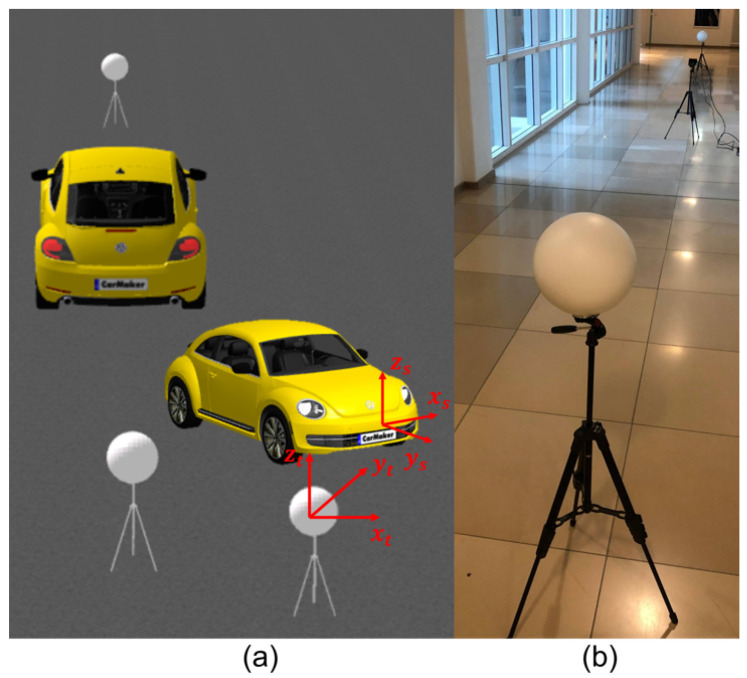
Measurement setup for the inside test. (**a**) Static simulation scene for the inside test. (**b**) Real static scene for the inside test. The sphere targets were placed at a distance of 6.7 m from the DUT in the simulation and real measurements. The reference distance $d_{ref}$ is calculated from the sensor’s origin to the target’s center. The coordinates of simulated and real objects and sensors are the same.

We have created a test setup in real and virtual environments, as shown in Figure 19, according to the specification given in Section 4.2. Cube 1 and the LiDAR FMU model use the same scan pattern with 500 scan lines and $0.5^{\circ }$ horizontal angle spacing, and the shape of the scan pattern is similar, as given in Figure 3. It should be noted that there is no restriction in the standard for the settings of the DUT, including FoV, the number of scan lines $N_{scans}$, and angular resolution $\theta_{res}$.

Before executing the inside test, we measured the diameter of the simulated and real sphere targets from the received LiDAR points, and the results are tabulated in Table 2. The results of the inside test are given in Table 3. We used the *osi3::GroundTruth* to retrieve the ground truth data in the simulation environment. The 3D objects are rendered in Blender 3D software [43] and integrated into the virtual environment of CarMaker.

The diameter ∅ of the sphere from the real and simulated LiDAR points can be estimated correctly, and the diameter error $\emptyset_{error}=\emptyset_{meas}-\emptyset_{ref}$ is negligible, as given in Table 2.

### 6.2. Symmetric Test

**Figure 20:**
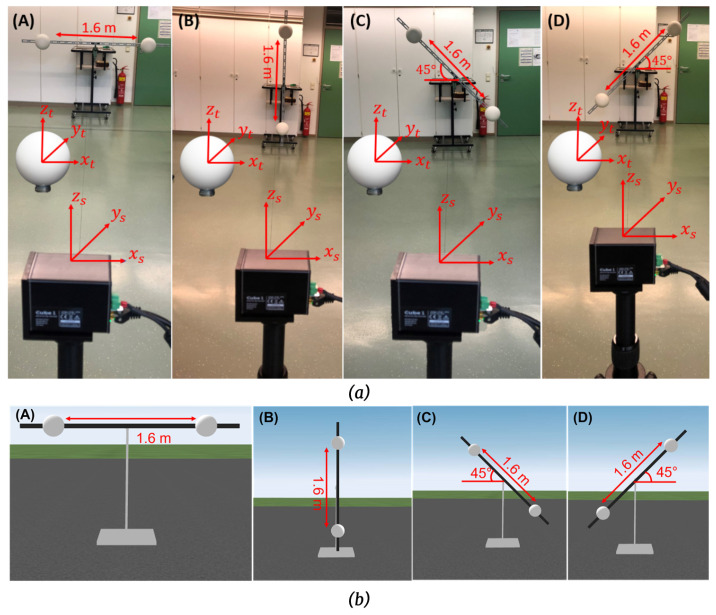
(**a**) Real test setup of symmetric tests for test positions (**A**–**D**). (**b**) Static simulation scenes of symmetric tests for test positions (**A**–**D**). The simulated and real sphere targets are placed in front of the sensor approximately at 5.5 m. The simulated and real bar length is 2 m long, while the distance between the sphere targets is 1.6 m. The coordinates of the simulated and actual objects and sensors are the same.

We have created the test setup according to the specifications defined in Section 4.3. The real and simulated test setups of the symmetric test are shown in Figure 20.

The DUT measures the two-sphere targets simultaneously placed in different orientations in this test. It should be noted that there is no restriction in the standard of the distance between the two sphere targets. We have chosen a 1.6 m distance between the sphere target because DUTs have a low vertical FoV. The LiDAR FMU and Cube 1 use the same scan pattern as the inside test, but the horizontal angle spacing is $0.4^{\circ }$. A total of eight measurements are taken in the front-sight mode, and all the tests are analyzed with an azimuth angle of $\theta =0^{\circ }$ between the DUT and the center line of the sphere targets because the second condition $\theta +90^{\circ }\pm 10^{\circ }$, as given in Section 4.3, is out of the DUTs work volume. The simulation and real results for the test positions A, B, C, and D are enumerated in Table 4.

### 6.3. Asymmetric Tests

**Figure 21:**
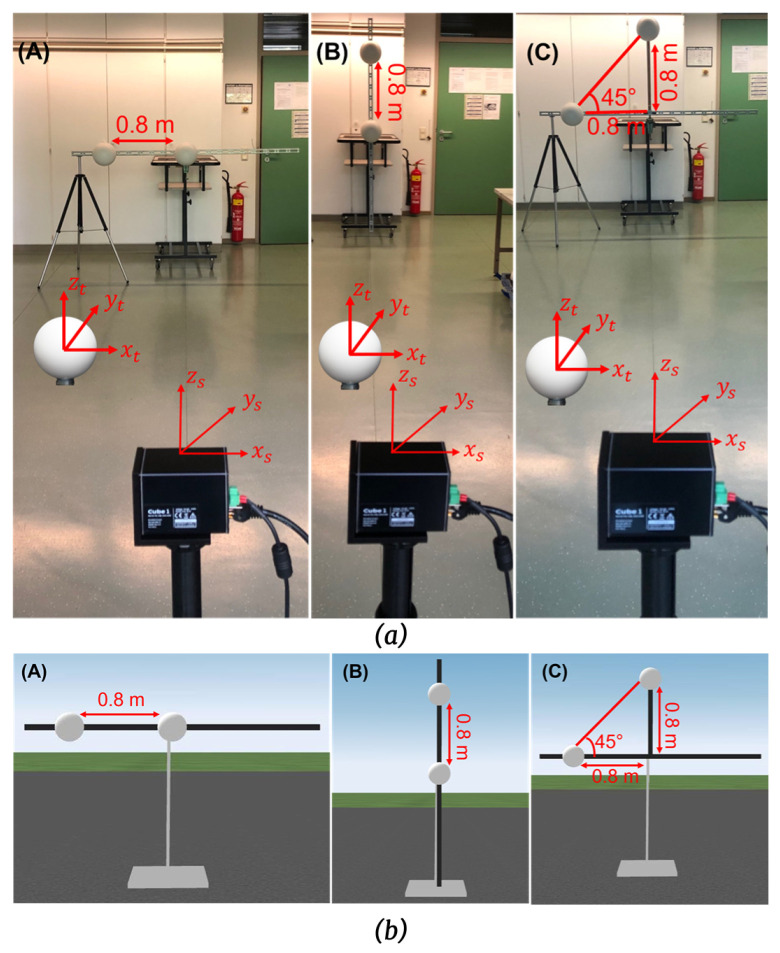
(**a**) Real test setup of asymmetric tests for test positions (**A**–**C**). (**b**) Static simulation scenes of asymmetric tests for test positions (**A**–**C**). The simulated and real sphere targets are placed in front of the sensor at approximately 5 m. The simulated and real bar length is 2 m long, while the distance between the sphere targets is 0.8 m. The coordinates of the simulated and actual objects and sensors are the same.

The real and simulation test setups were created to perform the asymmetric tests, as shown in Figure 21, according to the specifications given in Section 4.4.

We used an extra stand to ensure no movement in the steel bar compared to the previous tests. In addition, the sphere targets have magnet holding that makes them easy to fix on the steel bar. The distance between the sphere targets was 0.8 m for the asymmetric tests. Cube 1 and LiDAR FMU use the same scan pattern as symmetric tests for these tests. The simulation and real results for the asymmetric tests are enumerated in Table 5.

Cube 1 and the LiDAR FMU model pass inside, symmetric, and asymmetric tests because the number of received points $N_{points}$ from the sphere targets is more than 300, and the distance error $d_{error}$ is less than 20 mm. Furthermore, the real sensor and LiDAR FMU use the same scan pattern; the simulated and real objects’ sizes and orientations are also similar. That is why the number of received points $N_{points}$ obtained from the surface of actual and simulated spheres matches pretty well. However, the distance error $d_{error}$ of the real measurement is slightly higher than the simulation results for all the tests given above; a possible reason behind this deviation is an uncertainty in the reference measurement due to human error because it was very challenging to align the RI laser point to the center of the sphere target.

### 6.4. Relative Range Tests

**Figure 22:**
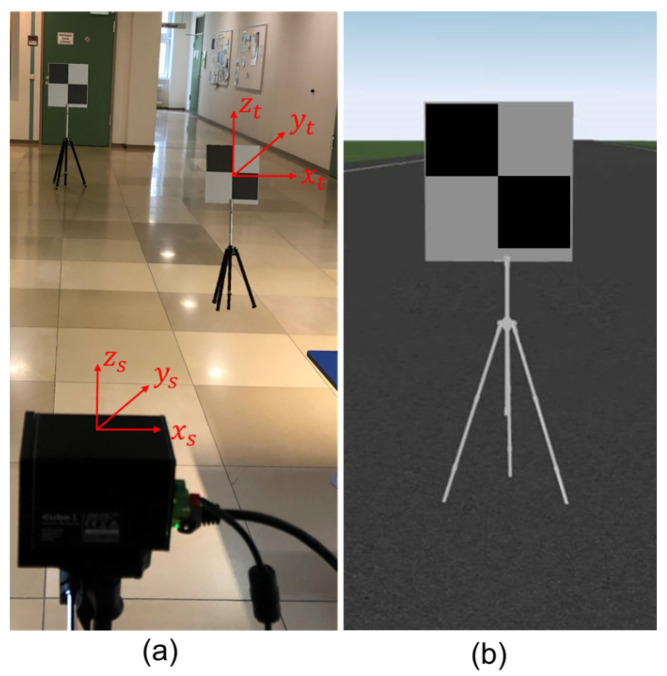
(**a**) Real setup for relative range tests. (**b**) Static simulation scene for relative range tests. The coordinates of the actual and simulated sensor and target are the same.

A test setup, as given in Section 4.5, was created to perform the relative range tests. The static simulation scene and real measurement setups are shown in Figure 22.

The reference distance $d_{ref}$ is calculated from the sensor origin to the center of the target. The reference position *A* was at 6 m, while three test positions, B, C, and D, were at 8, 9, and 10 m, respectively. The relative test positions from the reference position were $d_{AB}$ = 2 m, $d_{AC}$ = 3 m, and $d_{AD}$ = 4 m. The center of the DUTs and the fiducial point of the rectangular plate were collinear. The results of the relative range tests are given in Table 6.

Cube 1 and LiDAR FMU pass the relative range tests because the number of received points $N_{points}$ from the plate target is more than 100, and the distance error is below 20 mm. It should also be noted that the relative range test distance error $d_{error}$ is lower than the inside, symmetric, and asymmetric tests because it was easy to point the RI to the fiducial point of the plate target as compared to the sphere target.

### 6.5. Uncertainty Budget for ASTM E3125-17 Tests

This section provides the uncertainty budget for all the ASTM E3125-17 tests performed in real and virtual environments.

#### 6.5.1. Uncertainty Budget of Real Measurements

1. Contribution of RI (external influences): The RI has a range accuracy of ±1.0 mm, from 0.1 m to 10 m, with a confidence level of 95%. That is why we consider a 1.0 mm range uncertainty due to the RI for the ASTM E3125-17 tests because we place the targets within the 10 m.
2. Contribution of misalignment between the target and RI center (external influences): We aligned the center of the targets and the laser tracker of RI manually, and it is tough to always aim the laser tracker in the center of the sphere compared to the plate target. The highest standard uncertainty due to this factor was 3.9 mm for the top sphere of test position C of symmetric tests. However, for all the other tests, the standard uncertainty due to this factor is less than 3.9 mm.
3. Contribution of environmental conditions (external influences): All the tests were performed in the lab; therefore, environmental conditions’ influence on the measurements is negligible.
4. Contribution of DUT internal influences (internal influences): The ranging error $d_{error}$ due to the internal influences of the DUT is 5.4 mm for all the tests. These internal influences include the ranging error $d_{error}$ due to the internal reflection of the sensor, detector losses, peak detection algorithm, and precision loss due to the spherical coordinates conversion to the cartesian coordinates. It should be noted that the distance error $d_{error}$ due to the sensor’s internal influences may vary depending on the temperature (see point 3 above).

#### 6.5.2. Uncertainty Budget of Simulation

1. Contribution of DUT (internal influences): As given above, the LiDAR FMU simulation model considers the exact scan pattern, signal processing chain, and sensor-related effect of Blickfeld Cube 1. Therefore, the uncertainty due to the internal influences of the sensor model is 5.4 mm (see 4 above).
2. Contribution of environmental conditions effect model (external influences): Environmental conditions effects are not modeled for these tests.

### 6.6. Comparison of Simulation and Real Measurements Results

The simulation and real measurements show good qualitative agreement. Therefore, we use the mean absolute percentage error metric (MAPE) to quantify the difference between the simulation and real measurement results for all the tests described above.

(12) $$M=\frac{1}{n}\sum_{i=1}^{n}|\frac{y_{i}-x_{i}}{y_{i}}|,$$

where $y_{i}$ is the simulated value, the measured value is denoted by $x_{i}$, and *n* shows the total number of data points [44]. The MAPE for the point-to-point distance estimation *d* is 0.04%, and for the number of received points $N_{points}$, 1.4%.

### 6.7. User-Selected Tests

**Figure 23:**
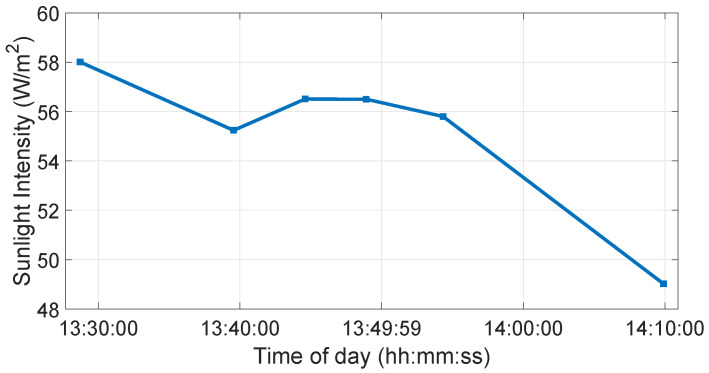
Sunlight intensity is measured on a cloudy day. The intensity of sunlight was recorded with an ADCMT 8230E optical power meter in W, and the sensor window size in m${}^{2}$ is used to calculate the sunlight intensity in W/m${}^{2}$.

**Figure 24:**
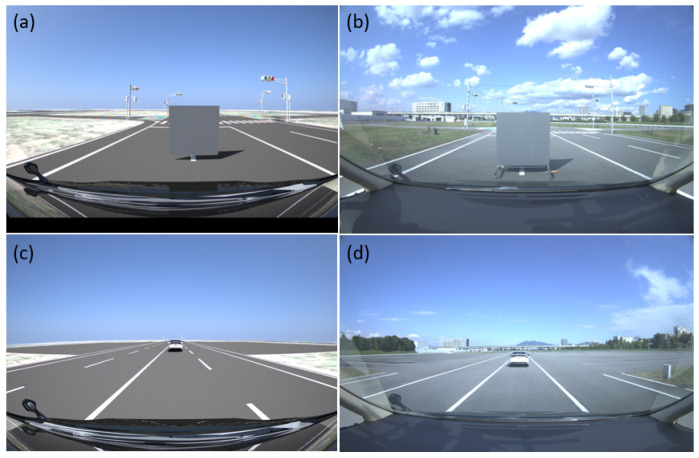
(**a**) Simulated static scene of plate target. (**b**) Static real scene of plate target. (**c**) Simulated static scene of vehicle target. (**d**) Real static scene of vehicle target. The ego vehicle is equipped with LiDAR, camera, and GNSS/INS RT3000 v3 from OxTS as a reference sensor with a range accuracy of 0.01 m. The LiDAR sensor was mounted on the vehicle’s roof, and the camera sensor was mounted on the front windscreen. The 10% reflective plate size is 1.5 × 1.5 m. The sensor position in the vehicle’s coordinates is *x* = 2279 mm, *y* = 96 mm, and *z* = 2000 mm. The reference distance is measured from the sensor’s reference point to the center of the Lambertian plate and the target vehicle trunk.

**Figure 25:**
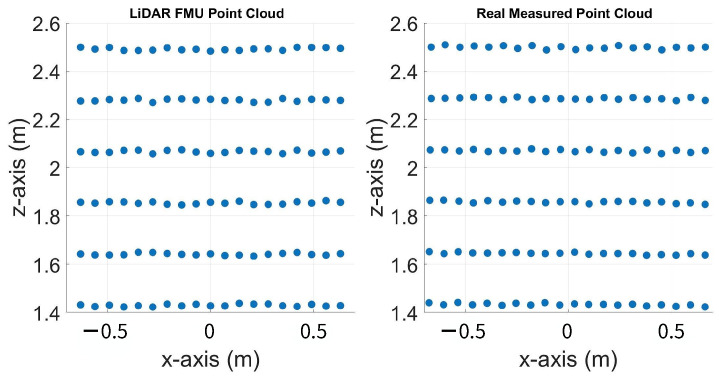
Visualization of LiDAR point clouds obtained from the real and simulated Lambertian plate placed at 20 m. We removed the LiDAR points from the edges of the plate for the data analysis, as recommended in the standard. Therefore, the effective area of the plate becomes 1.3 × 1.3 m.

**Figure 26:**
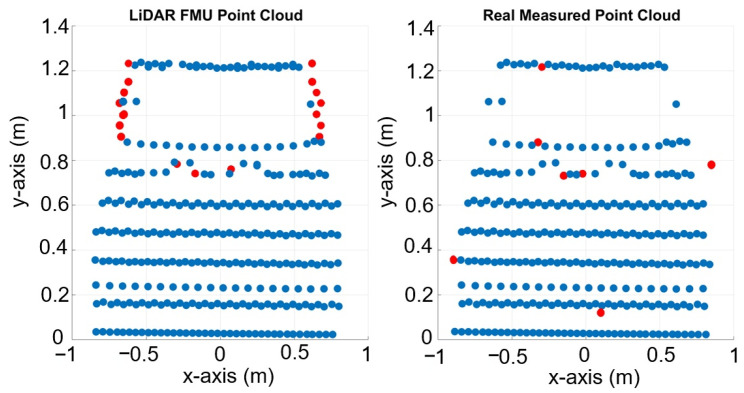
Visualization of LiDAR point clouds obtained from the real and simulated vehicle placed at 12 m. The actual width and height of the vehicle is 1.76 × 1.25 m, the LiDAR FMU and Cube 1 estimate 1.74 × 1.23 m and 1.74 × 1.22 m, respectively. The vehicle’s height is calculated from the bottom of the rear bumper to the vehicle’s roof. The red dots in the picture show the difference between the simulated and real point clouds.

**Figure 27:**
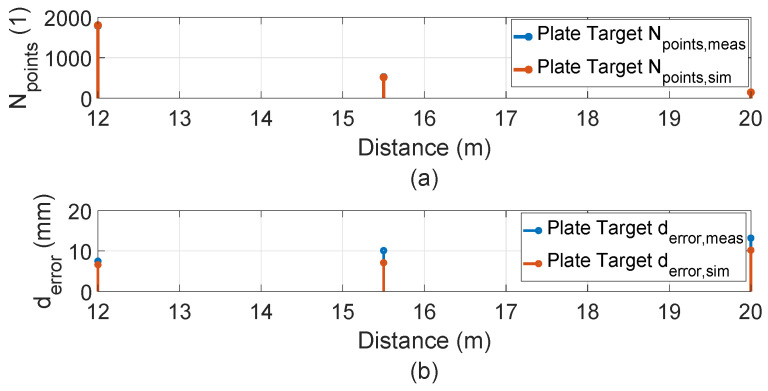
(**a**) Comparison of the number of points $N_{points}$ received from the surface of simulated and real 10% Lambertian plate. The simulation and real measurement results are consistent. (**b**) Comparison of real and virtual LiDAR sensor distance error $d_{error}$ for the plate target. The distance error $d_{error}$ is below MPE ± 20 mm.

**Figure 28:**
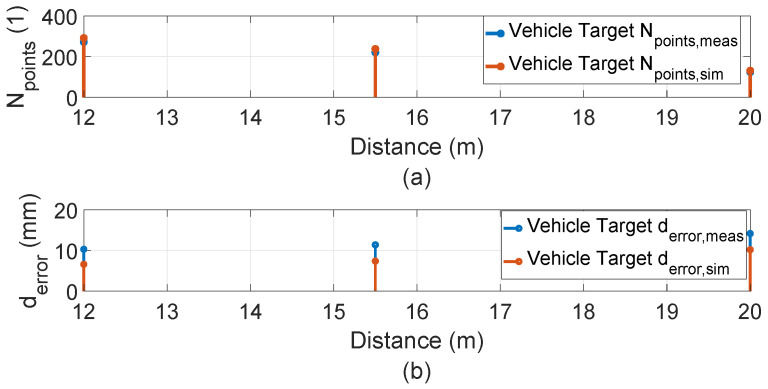
(**a**) Comparison of the number of points $N_{points}$ received from the surface of the simulated and real vehicle. (**b**) Comparison of the real and virtual LiDAR sensor distance error $d_{error}$ for the vehicle target. The distance error $d_{error}$ is below the MPE ±20 mm.

The inside, symmetric, asymmetric, and relative range tests were conducted in the lab. Therefore, we conducted several static tests to evaluate DUT measurement performance in sunlight at the proving ground. First, as shown in Figure 23, we recorded the daylight and modeled it in the LiDAR FMU [23]. We use a 10% reflective Lambertian plate and a Toyota Prius as targets for these tests, as shown in Figure 24. The ego and target vehicles were equipped with a global navigation satellite/inertial navigation system (GNSS/INS) for reference measurements. The scan pattern used by Cube 1 and LiDAR FMU for the user-defined tests is given in Figure 3. The exemplary LiDAR points $N_{points}$ received from the simulated and real Lambertian plate and vehicle targets are shown in Figure 25 and Figure 26. In addition, the simulation and real measurement results for the user-selected tests are given in Figure 27 and Figure 28.

The results show that the derived point-to-point distance estimation error $d_{error}$ of real and virtual LiDAR sensors is less than the MPE. However, the distance error $d_{error}$ is increased to 14.2 mm if the vehicle target is placed at a distance of 20 m in daylight. This is because the sunlight raises the background noise of the LiDAR signal, and point clouds become noisy and dispersed, shifting the derived point of the vehicle target. It should be noted that we use the manual estimation method to calculate the derived point on the trunk of the target vehicle. Furthermore, the dimensions of the objects can be estimated from the received real and virtual LiDAR point clouds. Moreover, the simulation and actual measurements show good agreement for the tests performed on the proving ground. For example, the MAPE for the number of received points $N_{points}$ is 3.8%, and for the distance *d*, is 0.04%. The simulation and real measurement results show a good correlation because the sensor model is developed with high fidelity, and real-world scenarios are replicated in the simulation with high accuracy by using the GNSS/INS data without manual interpolation.

### 6.8. Influence of ASTM Standard KPIs on Object Detection

Deep learning and neural networks are used for object recognition, segmentation, and classification from the 3D LiDAR point clouds. However, if the 3D imaging and position estimation of the LiDAR sensor degrades, it will influence the performance of the object recognition algorithm and ADAS. To show this and to ensure the usability of simulation models validated with the ASTM standard, we trained the state-of-the-art deep learning-based PointPillars network [45] for object detection using synthetic LiDAR data. Then, we tested it with real and simulated data of the vehicle target shown in Figure 26. We use the average orientation similarity (AOS) metric [46] to find the correlation between the ground truth 3D orientation of the object and the 3D orientation of the object estimated by the object detection algorithm. The object detection confidence score (ODCS) and average orientation similarity (AOS) of the real and simulated vehicle targets are given in Table 7.

**Figure 29:**
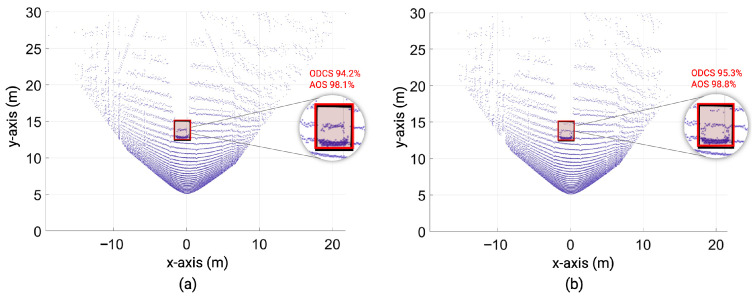
(**a**) Real point cloud data: Black and red cuboids represent the ground-truth 3D orientation of the object and the 3D orientation of the object estimated by the object detection algorithm, respectively. (**b**) Synthetic point cloud data: Black and red cuboids represent the ground-truth 3D orientation of the object and the 3D orientation of the object estimated by the object detection algorithm, respectively.

The results show that the ODCS of the PointPillar from simulated and real data is more than 90% up to 20 m. In addition, the AOS metric results are more than 98% for real and virtual LiDAR data because the object detection algorithm can correctly estimate the target dimensions and position from synthetic and real data, as shown in Figure 29.

**Figure 30:**
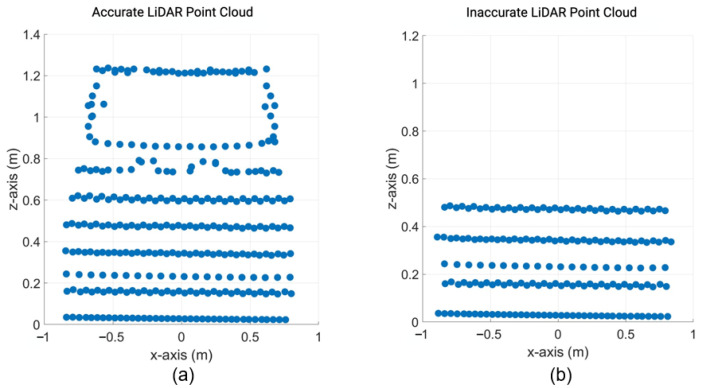
(**a**) Exemplary visualization of accurate LiDAR point cloud obtained from a simulated vehicle at 12.0 m. (**b**) Exemplary visualization of inaccurate LiDAR point cloud obtained from a simulated vehicle at 12.0 m. The actual width and height of the vehicle, 1.76 × 1.25 m, can not be estimated from the inaccurate data.

Furthermore, the simulation and real measurement also show good agreement because the MAPE for the number of received point clouds $N_{points}$ and the distance error $d_{error}$ is negligible. Afterward, we test the object detection algorithm with point clouds that do not meet the KPIs of the ASTM standard, as shown in Figure 30, to investigate the degradation in the performance of the object recognition algorithm. It should be noted that the LiDAR FMU vertical FoV is set in such a way so that the number of received points $N_{points}$ from the vehicle is decreased. The distance offset is also added intentionally to receive an inaccurate distance in the point cloud to see the impact of point-to-point distance estimation error $d_{error}$ on the performance of the object recognition algorithm.

**Figure 31:**
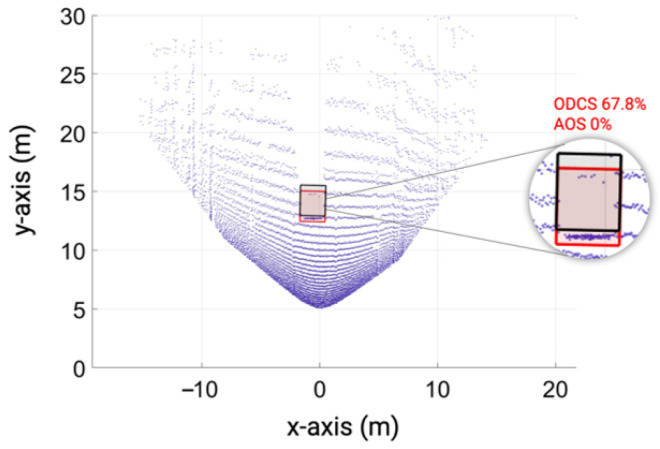
Exemplary visualization of inaccurate simulated point cloud data: The object detection score drops to 67.8% from 95.3%, and a −0.8 m offset in position leads to a shift in the 3D bounding box of the object predicted by the object detection algorithm, shown with the red color cuboid. The black cuboid shows the ground-truth 3D orientation of the object.

Figure 31 shows that if the object recognition algorithm can not estimate the shape or size of the object from the given point clouds, it will degrade its confidence about the object’s existence. In addition, if the point-to-point distance or 3D position estimation performance of the LiDAR sensor degrades, then the object detection algorithm will detect the target at the wrong position. These two key performance indicators are critical in evaluating the measurement performance of automotive LiDAR sensors. Therefore, initial results show that the specification and test frameworks defined in the ASTM E3125-17 standard can be used to evaluate the measurement performance of virtual and real automotive LiDAR sensors, at least at the early stage of development, because the standard consists of static scenarios that can be implemented easily in real and virtual environments.

## 7. Conclusions

In this work, we evaluated the point-to-point distance measurement performance of the MEMS-based automotive LiDAR sensor Cube 1 from Blickfeld and its simulation model according to the ASTM E3125-17. The LiDAR simulation model is developed using the standardized interfaces OSI and FMI. It considers the accurate modeling of the scan pattern and complete signal processing steps of Blickfeld Cube 1. The ASTM E3125-17 standard defines the specifications and static test cases to evaluate the measurement performance of the LiDAR sensors.

The virtual and real automotive LiDAR sensor passes all the tests defined in the standard because the point-to-point distance estimation error $d_{error}$ is less than MPE, and the size of the objects can be estimated correctly from the received point clouds. The simulation and real measurements show good agreement. For example, for the ASTM tests, the MAPE of the number of points $N_{points}$ and point-to-point distance estimation *d* of the virtual and real targets are 1.4% and 0.04%.

In addition, we also performed measurements at the Jtown proving ground for verifying DUT’s operational performance in daylight conditions. However, the point-to-point distance error $d_{error}$ is less than MPE. Furthermore, the object’s dimensions and size can be estimated correctly from the received points. The real-world scenarios and environmental conditions are replicated in the virtual environment to obtain the synthetic data. The MAPE for the actual and simulation data is 3.8% for the number of received points $N_{points}$ and 0.04% for the distance estimation *d*. It should be noted that it is very challenging to model 100% real-world environmental conditions in the virtual environment, which is why the MAPE of user-defined tests for the $N_{points}$ is increased to 3.8% from 1.4%.

To realize the effect of the MAPE of the simulation and real measurement for the KPIs defined in ASTM E3125-17 standard on object recognition, we trained a deep learning-based PointPillars network by using the simulated point cloud data and testing it with the real and simulated point clouds. The simulated and real point clouds also show good agreement at the object level; for instance, the MAPE for ODCS and AOS of the simulated and real point cloud is 2.0% and 1.0%, respectively. If the LiDAR sensor 3D imaging and position estimation performance drop, it influences the performance of the object detection algorithm. For instance, a 0.8 m distance error $d_{error}$ will lead the AOS from 98.8% to 0%.

Therefore, it is concluded that virtual and real LiDAR sensors work for their intended use, at least for static use cases. Furthermore, the 3D imaging and point-to-point distance estimation capability are critical to evaluate the operational performance of automotive virtual and real LiDAR sensors. That is why it can also be concluded from the initial results that the specifications and test framework defined in ASTM E3125-17 can be used to evaluate the measurement performance of automotive virtual and real LiDAR sensors at the early stages of development.

## 8. Outlook

All the tests defined in ASTM E3125-17 are static. Furthermore, no standardized tests are available to evaluate the sensor performance in dynamic tests. That is why, in the next steps, a set of dynamic test cases will be defined from expert knowledge to assess the 3D imaging, point-to-point distance estimation, angular resolution, range resolution, and range accuracy measurement performance of real and virtual LiDAR sensor performance. Moreover, we will further investigate the impact of these KPIs of the LiDAR sensor on the performance of the object recognition algorithm.

## Figures and Tables

**Table 1:** Parameter specification of Cube 1 LiDAR sensor [28].

Parameters	Values
Typical application range	1.5 m–75 m
Range resolution	<1 cm
Range precision (bias-free RMS, 10 m, 50% reflective target). The standard deviation of range precision is one σ, which means a coverage of 68.26% [34].	<2 cm
FoV (H × V)	$70^{\circ }$ × $30^{\circ }$
Horizontal resolution	$0.4^{\circ }$–$1^{\circ }$ (user configurable)
Vertical resolution	5–400 scan lines per frame (user configurable)
Frame rate	1.5 Hz–50 Hz (user configurable)
Laser wavelength	905 nm

**Table 2:** Diameter ∅ and the cartesian coordinates of derived points of simulated and real sphere targets are obtained by LSSF.

	x (mm)	y (mm)	z (mm)	Reference Diameter $\emptyset_{\mathrm{ref}}$ (mm)	Measured Diameter $\emptyset_{\mathrm{meas}}$ (mm)	Diameter Error $\emptyset_{\mathrm{error}}$ (mm)
Cube 1	1.3	6672.1	125.5	200.9	201.2	0.3
LiDAR FMU	2.1	6672.4	121.2	200.0	200.1	0.1

**Table 3:** Inside test results.

	Target	No. of Points (1)	Reference Distance to Target $d_{\mathrm{ref}}$ (mm)	Measured Distance $d_{\mathrm{meas}}$ (mm)	Distance Error $d_{\mathrm{error}}$ (mm)	MPE (mm)	Pass/Fail
Cube 1	Front sphere	354	6680.0	6689.0	9.0	20.0	Pass
Cube 1	Back sphere	358	6680.0	6686.3	6.3	20.0	Pass
LiDAR FMU	Front sphere	358	6680.0	6685.5	5.5	20.0	Pass
LiDAR FMU	Back sphere	358	6680.0	6685.5	5.5	20.0	Pass

**Table 4:** Results of symmetric tests.

	Test Position	Target	No. of Points (1)	Reference Distance to Target $d_{\mathrm{ref}}$ (mm)	Measured Distance $d_{\mathrm{meas}}$ (mm)	Distance Error $d_{\mathrm{error}}$ (mm)	MPE (mm)	Pass/Fail
Cube 1	A	Left sphere	326	5050.0	5056.2	6.2	20.0	Pass
Cube 1	A	Right sphere	319	5050.0	5059.1	9.1	20.0	Pass
LiDAR FMU	A	Left sphere	327	5050.0	5055.7	5.7	20.0	Pass
LiDAR FMU	A	Right sphere	327	5050.0	5055.7	5.7	20.0	Pass
Cube 1	B	Top sphere	321	5050.0	5058.2	8.2	20.0	Pass
Cube 1	B	Bottom sphere	325	5050.0	5057.3	7.3	20.0	Pass
LiDAR FMU	B	Top sphere	322	5050.0	5055.9	5.9	20.0	Pass
LiDAR FMU	B	Bottom sphere	323	5050.0	5055.8	5.8	20.0	Pass
Cube 1	C	Top sphere	338	5050.0	5059.3	9.3	20.0	Pass
Cube 1	C	Bottom sphere	343	5050.0	5058.8	8.8	20.0	Pass
LiDAR FMU	C	Top sphere	340	5050.0	5055.6	5.6	20.0	Pass
LiDAR FMU	C	Bottom sphere	339	5050.0	5055.8	5.8	20.0	Pass
Cube 1	D	Top sphere	333	5050.0	5058.1	8.1	20.0	Pass
Cube 1	D	Bottom sphere	332	5050.0	5057.8	7.8	20.0	Pass
LiDAR FMU	D	Top sphere	336	5050.0	5055.4	5.4	20.0	Pass
LiDAR FMU	D	Bottom sphere	338	5050.0	5055.2	5.2	20.0	Pass

**Table 5:** Results of asymmetric tests.

	Test Position	Target	No. of Points (1)	Reference Distance to Target $d_{\mathrm{ref}}$ (mm)	Measured Distance $d_{\mathrm{meas}}$ (mm)	Distance Error $d_{\mathrm{error}}$ (mm)	MPE (mm)	Pass/Fail
Cube 1	A	Center sphere	332	5050.0	5057.7	7.7	20	Pass
Cube 1	A	Left sphere	323	5050.0	5058.3	8.3	20	Pass
LiDAR FMU	A	Center sphere	339	5050.0	5055.7	5.7	20	Pass
LiDAR FMU	A	Left sphere	325	5050.0	5055.9	5.9	20	Pass
Cube 1	B	Top sphere	319	5050.0	5058.2	8.2	20	Pass
Cube 1	B	Center sphere	328	5000.0	5006.9	6.9	20	Pass
LiDAR FMU	B	Top sphere	323	5050.0	5055.5	5.5	20	Pass
LiDAR FMU	B	Center sphere	331	5000.0	5005.4	5.4	20	Pass
Cube 1	C	Top sphere	317	5000.0	5008.8	8.8	20	Pass
Cube 1	C	left sphere	322	5050.0	5057.4	7.4	20	Pass
LiDAR FMU	C	Top sphere	323	5000.0	5005.7	5.7	20	Pass
LiDAR FMU	C	Left sphere	324	5050.0	5055.5	5.5	20	Pass

**Table 6:** Results of relative range tests.

	Target Position	No. of Points (1)	Reference Distance to Target $d_{\mathrm{ref}}$ (mm)	Measured Distance $d_{\mathrm{meas}}$ (mm)	Distance Error $d_{\mathrm{error}}$ (mm)	MPE (mm)	$q_{\mathrm{rms}}$ (mm)	Pass/Fail
Cube 1	AB	451	2000.0	2005.7	5.7	20	1.5	Pass
Cube 1	AC	290	3000.0	3005.7	5.7	20	1.7	Pass
Cube 1	AD	208	4000.0	4007.3	7.3	20	1.8	Pass
LiDAR FMU	AB	462	2000.0	2005.5	5.5	20	1.1	Pass
LiDAR FMU	AC	298	3000.0	3005.5	5.4	20	0.6	Pass
LiDAR FMU	AD	217	4000.0	4005.5	5.4	20	0.3	Pass

**Table 7:** Results of the object detection algorithm for simulated and real data.

	Target	Distance (m)	ODCS (%)	AOS (%)
Cube 1	Vehicle	12.0	94.2	98.1
Cube 1	Vehicle	15.5	92.8	97.7
Cube 1	Vehicle	20.0	90.6	97.2
LiDAR FMU	Vehicle	12.0	95.3	98.8
LiDAR FMU	Vehicle	15.5	94.6	98.6
LiDAR FMU	Vehicle	20.0	93.3	98.5

## Data Availability

Not applicable.

## Abbreviations

The following abbreviations are used in this manuscript:

ACC	Adaptive cruise control
AOS	Average orientation similarity
ADAS	Advanced driver-assistance system
BSD	Blind-spot detection
DUT	Device under test
Effect Engine	FX engine
FMU	Functional mock-up unit
FMI	Functional mock-up interface
FCW	Forward collision warning
FoV	Field of view
GNSS	Global navigation satellite system
INS	Inertial navigation system
LDWS	Lane departure warning system
LiDAR	Light detection and ranging
LDM	Laser and detector module
LSSF	Least square sphere fit
LSPF	Least square plane fit
MPE	Maximum permissible error
MEMS	Micro-electro-mechanical systems
MAPE	Mean absolute percentage error
OSI	Open simulation interface
OEMs	Original equipment manufacturers
OSMP	OSI sensor model packaging
OPA	Optical phased array
ODCS	Object detection confidence score
RADAR	Radio detection and ranging
RTDT	Round-trip delay time
RI	Reference instrument
RMS	Root mean square
